# Annotations

**Published:** 1903-05-23

**Authors:** 


### Annotations.

### Small-pox and the Colonial Office.

The accounts received from various sources as to the public health in the West Indies are curious and in many respects not very pleasant reading. About a year ago the island of Barbados notified some cases of small-pox which had been imported from Halifax. It was the same mild type of disease which was very prevalent in the North of America in 1900-1 and in Barbados was actively taken in hand. It spread however with some steadiness, so that although the death-rate was very low, only about 7 per cent., the number of cases was considerable, *i.e.*, about 1,500. During all this time Barbados was subjected to the severest form of quarantine by all its neighbours and in consequence of this suffered very heavy trade losses. Barbados' loss was Trinidad's gain, and smarting under the knowledge of this fact, a sharp look-out was kept on the health of Trinidad by Barbados. Presently it received word that many cases of an eruptive infectious disorder had occurred in Trinidad, and though they were diagnosed under various strange terms, such as Carupano-pox, and varioloid varicella, they seemed to Barbados to be nothing more nor less than the same disease through which Barbados itself had been made to suffer so severely. Thereupon Barbados "made remarks," and in consequence was invited to come and look for itself. This it did in the person of a Dr. Bridger. The latter reported that the hospitals of Trinidad were beginning to overflow with cases; that the disease was small-pox and nothing else; and that nothing was being done to arrest it. Trinidad however stoutly refused to admit that the disease was anything more than chicken-pox, and thereon an island internecine warfare set in with quarantine for a weapon. Trinidad continued to issue clean bills of health, and various islands for a time took various sides; but at length all have combined to put Trinidad into quarantine. Meantime, however, there is reason to suppose that British Guiana and Grenada have both become infected with the same disease. There seems, therefore, every probability that a long epidemic of small-pox is likely to be added to the general troubles of all the West Indian colonies. A further unpleasant feature of the matter is that there seems to have been a distinct attempt on the part of the Trinidad authorities to burke facts in the supposed interests of Trade. What has the Colonial Office to say in the matter? A question might usefully be put to the Colonial Secretary.

### Infection in the Electric Trams.

There was an interesting inauguration, on Friday, 15th inst., of a system of electrical tramways from Westminster, Waterloo, and Blackfriars to Tooting. The Prince of Wales having declared the line open, he and the Princess, with the two young Princes, travelled in the first of the cars from Westminster to Tooting and back. The new cars easily accommodate 28 passengers inside and 32 outside, are admirably designed, ventilated, and lighted. In order to mitigate, as far as possible, the annoyance of non-smokers on the outside from the fumes of tobacco, smokers are requested to take seats in the rear, and with the view of preventing the disgusting and dangerous habit of spitting on the floor, it is intimated that any person guilty of the offence will be proceeded against. This last very desirable, if somewhat drastic, course, is in curious contrast to the encouragement afforded to the propagation of infectious diseases, and of things hardly less objectionable, by the upholstered seats provided inside the cars under the auspices of the London County Council. Instead of being content with plain wood or cane they have covered the seats with thick mats of much the same texture as Axminster carpet. While the cars are new the appearance of these covered seats may be more pleasing, but when they have been in use for a little time it will be impossible to urge even that plea in their favour. We cannot think why the County Council spent such an unnecessary amount of the ratepayers' money on the purchase of material which is not wanted for comfort, conduces to uncleanliness, and must be a constant source of peril to health. However, little mischief can have been done at present, and we are hopeful that now attention has been called to the insanitary nature of the covered seats in the cars they will soon be relieved of their superfluous and germ-carrying mats.

### A Chance for Inventors.

This is the age of competition and competitions, and the latest one published is fathered by the Society of Arts. Naturally, therefore, it has a more useful and serious purpose than most of its forbears. The prize offered is a gold medal or the sum of £20. This will go to the competitor who, on or before the last day of this year, sends in the most useful specimen of a respirator for the use of workers engaged in occupations which give rise to a dangerous amount of dust, whether organic or inorganic. The desiderata are lightness, simplicity, cheapness of manufacture, capability of having the filtering medium easily removed and replaced, that the instrument shall allow air to enter neither mouth or nostrils without passing through the filtering medium, that it shall not give rise to rebreathing of respired air, and finally that it shall not be unpleasant to wear during hours of hard work. It is more than eighty years ago since the Society of Arts awarded its gold medal to the inventor of a respirator designed to diminish the risks of Sheffield Dry Grinding. The virtue of this respirator depended upon the use of magnets. It was quite a practical contrivance, but it did not "catch on" for a somewhat pathetic reason. The workers for whose benefit it was designed would not wear it because they were afraid that if the dangers of their trade were diminished their pay might be cut down. One factor in the case probably was that workers in the dangerous trades get too used to risks or are too blind to them to be readily persuaded to adopt precautions which give them any trouble. It is difficult to get them to use the simplest precautions, and the only real remedy is to organise the work in such fashion that those employed do not depend upon their own caution for their well-being while engaged in it. Little by little, and under the influence of the Home Office, this is being done in nearly all the dangerous trades. There are special difficulties in the dusty trades, but they will be got over in the course of time.

